# Molecular authentication of *Cissampelos pareira* L. var. *hirsuta* (Buch.-Ham. ex DC.) Forman, the genuine source plant of ayurvedic raw drug ‘*Patha*’, and its other source plants by ISSR markers

**DOI:** 10.1007/s13205-013-0183-8

**Published:** 2013-11-26

**Authors:** Deepu Vijayan, Archana Cheethaparambil, Geetha Sivadasan Pillai, Indira Balachandran

**Affiliations:** 1 Crop Improvement and Biotechnology Division, Centre for Medicinal Plants Research, Arya Vaidya Sala, Kottakkal, Malappuram, 676503 Kerala India; 2 Present Address: Botanical Survey of India, Eastern Regional Centre, Shillong, 793003 India

**Keywords:** Adulteration, Substitution, Molecular markers, *Cyclea peltata*, *Stephania japonica*

## Abstract

*Cissampelos pareira* L. var. *hirsuta* (Buch.-Ham. ex DC.) Forman belongs to family Menispermaceae. The roots of this taxon are used in the treatment of various diseases like stomach pain, fever, skin disease, etc., in Ayurveda and is commonly known as *Patha*. Two other species, viz., *Cyclea peltata* (Lam.) Hook.f. & Thomson and *Stephania japonica* (Thunb.) Miers of the same family are being used as the source of this drug in various parts of India. This type of substitution or adulteration will ultimately affect the therapeutic efficacy of the medicines adversely. ISSR profiles of all the three taxa are generated and analyzed to assess the genetic relationships among these three species. The profiles of all the three species displayed a high level of polymorphism among them. ISSR markers developed can be used in authenticating and validating the exact species discrimination of the genuine raw drug of ‘*Patha*’ from its substitutes/adulterants to guarantee the quality and legitimacy of this drug in the market.

## Introduction

The roots of *Cissampelos pareira* L. var. *hirsuta* (Buch.-Ham. ex DC.) Forman, *Cyclea peltata* (Lam.) Hook.f. & Thomson and *Stephania japonica* (Thunb.) Miers of family Menispermaceae are known as *Patha* in Ayurveda. They are used in the treatment of various diseases like stomach pain, fever, skin conditions, cardiac pain, etc., among which *C. pareira* var. *hirsuta* is the accepted source in Ayurveda (API 2001; Yoganarsimhan 1996). However, the plants of *C. peltata* are used as *Patha* in Kerala (Warrier et al. 1994). Authentication of raw medicinal plants is a fundamental requirement for quality assurance in herbal drug markets. Certain rare and expensive medicinal plant species are often adulterated or substituted by morphologically similar, easily available or less expensive species. Pharmaceutical companies procure plant materials from traders, who gather them from untrained collectors in the rural and forest areas. This has given rise to widespread adulteration or substitution, leading to poor quality of herbal formulations (Mehrotra and Rawat 2000). Herbal medicinal products may vary in composition and properties, unlike conventional pharmaceutical products, which are usually prepared from synthetic, chemically pure materials by means of reproducible manufacturing techniques and procedures. Correct identification and quality assurance of the starting material is, therefore, an essential prerequisite to ensure reproducible quality of herbal medicine, which contributes to its safety and efficacy (De Smet 2002; Straus 2002).

The morphological, biochemical or histological characteristics employed in the identification are prone to different environmental conditions (Kiran et al. 2010). Limitations of these markers for authentication of herbal drugs have generated a need to develop more reproducible molecular markers for quality control of these medicinal herbs. In view of these limitations, there is need for a new approach that can complement or, in certain situations, serve as an alternative. DNA markers are reliable for informative polymorphisms as the genetic composition is unique for each species and is not affected by age, physiological conditions as well as environmental factors (Chan 2003). DNA-based molecular markers have proved their utility in various fields of science and recently researchers have tried to explore the application of these markers in pharmacognostic characterization of herbal medicine. DNA-based techniques have been widely used for authentication of plant species of medicinal importance. This is especially useful in case of those that are frequently substituted or adulterated with other species or varieties that are morphologically and/or phytochemically indistinguishable. Attempts have been made to compare the source plants of *Patha* using anatomical and phytochemical markers (Hullatti and Sharada 2007, 2010). But, so far nobody has attempted to characterize these three species at the molecular level. The main objective of the present study is to evaluate the source plants of *Patha* using inter simple sequence repeat (ISSR) markers for the accurate identification of *C. pareira* var. *hirsuta, C. peltata* and *S. japonica*.

## Materials and methods

### Plant materials and DNA extraction

The total genomic DNA was extracted from the fresh leaves of *C. pareira* var. *hirsuta*, *C. peltata* and *S. japonica* grown at the Centre for Medicinal Plants Research, Arya Vaidya Sala, Kottakkal, Kerala, India, using a modified CTAB method (Doyle and Doyle 1987). The leaf samples (0.2 g) were ground in liquid nitrogen using mortar and pestle and re-suspended in 2 mL of DNA extraction buffer [2 % CTAB, 1 % PVP, 1.4 M NaCl, 20 mM EDTA (pH 8.0), 100 mM Tris–HCl (pH 8.0) and 0.2 % β-mercaptoethanol]. The mixture was incubated at 65 °C for 30 min and centrifuged with equal volume of chloroform:isoamylalcohol (24:1) at 8,000 rpm for 10 min. The supernatant was transferred to fresh tubes and DNA was precipitated by adding equal volume of ice cold isopropanol. The DNA precipitate was washed with 70 % ethanol, air dried and stored in 500 μL TE buffer. RNA was eliminated by treating the samples with RNase A (10 mg/mL) at 37 °C for 30 min. Again, it was extracted with equal volume of chloroform:isoamylalcohol (24:1). Two volumes of cold ethanol were added in aqueous layer and centrifuged at 12,000 rpm for 10 min. Pellets were washed with 70 % alcohol, air dried and dissolved in TE buffer. DNA quantification as well as quality assessment was carried out spectrophotometrically using Biophotometer (Eppendorf—AG 22331, Germany). The DNA sample was also quantified on 0.8 % agarose gel electrophoresis.

### ISSR-PCR

Nineteen ISSR primers (Eurofins, Bangalore, India) were selected for the present investigation. PCR for amplifying the DNA preparations was carried out in a 25-μl volume of reaction mixture. A reaction tube contained 25 ng of DNA, 1 U of *Taq* DNA polymerase enzyme, 2.5 mM of each dNTPs, 1× *Taq* buffer with 25 mM MgCl_2_ and 25 pmol primers. Amplifications were carried out in a DNA thermal cycler (Eppendorf, mastercycler gradient) using following parameters: 94 °C for 5 min; 35 cycles at 94 °C for 1 min, 49–59 °C for 1 min, and 72 °C for 2 min; and a final extension at 72 °C for 10 min. The annealing temperature was adjusted to a range of 49–59 °C depending on GC content and length of the primers. PCR products were subjected to agarose gel [1.5 % (w/v)] electrophoresis in 1× TBE buffer, along with 1 kb DNA ladder (Fermentas Life Sciences) as size markers. DNA was stained with ethidium bromide and electrophoretic profile was photographed on gel documentation system (Alpha Innotech, USA).

## Results and discussion

**Fig. 1:**
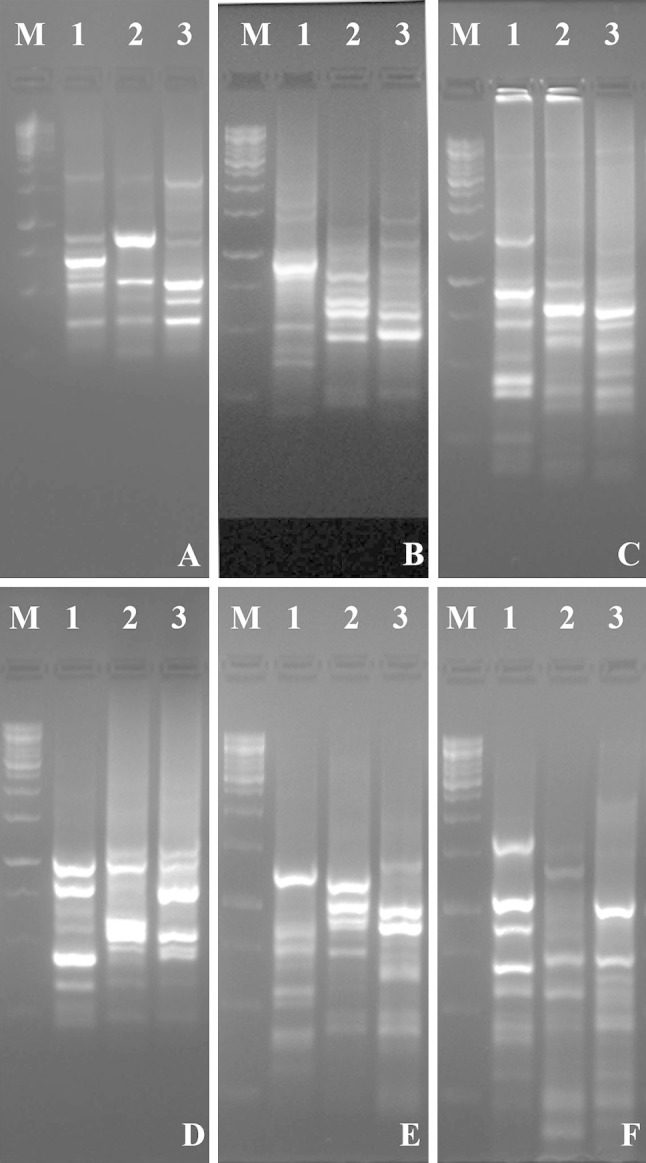
Representative ISSR profiles of *Patha* plants using primers **a** (ACTG)_4_, **b** UBC 840, **c** UBC 847, **d** UBC 857, **e** UBC 881 and **f** UBC 890. *M* represents 1 kb ladder, *lane 1Cissampelos pareira* var. *hirsuta*, *lane 2Cyclea peltata* and *lane 3Stephania japonica*

ISSR fingerprinting of 19 primers generated 270 scorable fragments, out of which 252 were polymorphic (93.33 % polymorphism) (Table 1). The primers viz., UBC 808, 810, 840, 841, 855, 862, 881 and 900 exhibited 100 percent polymorphism. The number of polymorphic fragments for each primer varied from 7 to 19 with an average of 13.26 polymorphic fragments (Fig. 1). ISSR banding pattern exhibited a maximum of 133 fragments in *S. japonica* followed by *C. pareira* var. *hirsuta* (132 fragments) and *C. peltata* (119). A maximum of eight unique bands were observed in *C. pareira* var. *hirsuta* while screening with the primer UBC 851. Interestingly, the number of unique bands in *C. peltata* was less compared to other plants. But in *S. japonica*, six and seven unique bands were observed while screening with the primers UBC 890 and UBC 900, respectively. Here also, the total number of unique bands was highest in *C. pareira* var. *hirsuta* (Table 2).

**Table 1:** Total number of bands and percentage of polymorphism amplified by 19 ISSR primers

Sl. no.	Primer	Annealing temperature	Total number of bands	Number of polymorphic bands	Number of monomorphic bands	Polymorphism (%)
1	(ACTG)_4_	49	11	10	1	90.91
2	UBC 807	50	9	8	1	88.89
3	UBC 808	52	17	17	0	100
4	UBC 810	50	16	16	0	100
5	UBC 825	50	14	13	1	92.86
6	UBC 840	54	17	17	0	100
7	UBC 841	54	12	12	0	100
8	UBC 842	54	10	8	2	80
9	UBC 847	52	16	13	3	81.25
10	UBC 851	54	16	14	2	87.5
11	UBC 854	56	13	12	1	92.31
12	UBC 855	52	19	19	0	100
13	UBC 857	54	15	14	1	93.33
14	UBC 859	54	8	7	1	87.50
15	UBC 862	55	13	13	0	100
16	UBC 873	52	12	9	3	75
17	UBC 881	59	19	19	0	100
18	UBC 890	59	20	18	2	90
19	UBC 900	59	13	13	0	100
		Total	270	252	18	93.33

**Table 2:** Primerwise ISSR banding pattern of *Patha* plants

Primer	*Cissampelos pareira* var. *hirsuta*				*Cyclea peltata*				*Stephania japonica*			
	TB	PB	MB	UB	TB	PB	MB	UB	TB	PB	MB	UB
(ACTG)_4_	6	5	1	3	4	3	1	1	6	5	1	3
UBC 807	4	3	1	0	7	6	1	4	3	2	1	1
UBC 808	9	9	0	5	4	4	0	1	9	9	0	5
UBC 810	7	7	0	4	10	10	0	5	6	6	0	0
UBC 825	9	8	1	6	6	5	1	1	6	5	1	1
UBC 840	9	9	0	3	10	10	0	3	7	7	0	2
UBC 841	3	3	0	2	4	4	0	4	6	6	0	5
UBC 842	4	2	2	0	7	5	2	2	7	5	2	2
UBC 847	9	6	3	5	7	4	3	2	9	6	3	3
UBC 851	11	9	2	8	7	5	2	1	6	4	2	1
UBC 854	5	4	1	2	7	6	1	3	6	5	1	4
UBC 855	9	9	0	7	6	6	0	1	10	10	0	5
UBC 857	9	8	1	5	7	6	1	1	8	7	1	1
UBC 859	4	3	1	3	4	3	1	1	4	3	1	1
UBC 862	6	6	0	4	6	6	0	3	5	5	0	2
UBC 873	7	4	3	4	5	2	3	2	6	3	3	3
UBC 881	9	9	0	6	8	8	0	3	9	9	0	3
UBC 890	9	7	2	5	7	5	2	3	12	10	2	6
UBC 900	3	3	0	3	3	3	0	2	8	8	0	7
Total	132	114	18	75	119	101	18	43	133	115	18	55

*TB* number of total bands, *PB* number of polymorphic bands, *MB* number of monomorphic bands, *UB* number of unique bands

Morpho-anatomical studies of roots of *C. pareira* var. *hirsuta*, *C. peltata* and *S. japonica* clearly indicate the significant differences among themselves (Hullatti and Sharada 2007). Hullatti and Sharada (2010) reported the HPTLC and HPLC fingerprinting of *Patha* plants and their studies clearly indicate the significant differences among the three plant materials. They concluded that all the studied parameters clearly indicate that roots of *C. pareira* var. *hirsuta* are the genuine source of *Patha* as it fulfills the ayurvedic claims of this drug. The present investigation confirms that there is no genetic relationship among *C. pareira* var. *hirsuta*, *C. peltata* and *S. japonica.* The high level of polymorphism and unique fragments detected in ISSR profiles authenticate that there exists a wide genetic disparity among the selected plants. The findings of the present study substantiate the reports on the applicability of ISSR markers to authenticate the herbal medicinal materials from its adulterants (Shen et al. 2006; Wang 2011). This investigation is the first report on the study of genetic relationships of *Patha* group of plants using ISSR markers.

## Abbreviations

API: Ayurvedic Pharmacopoeia of India
CTAB: Cetyl trimethyl ammonium bromide
DNA: Deoxyribonucleic acid
ISSR: Inter simple sequence repeats
PCR: Polymerase chain reaction
